# Enhancing understanding of healthy aging based on time-varying dependencies among multidimensional health, life satisfaction, and health behaviors of older adults aged 60 years and over

**DOI:** 10.1186/s12889-024-17752-2

**Published:** 2024-01-16

**Authors:** Jianghua Zhang, Yunbao Zhang, Zhiyi Wu, Xuemei Fu

**Affiliations:** School of Management, Shandong University, Jinan, 250100 China

**Keywords:** Multidimensional health, Health behaviors, Life satisfaction, Time-varying dependencies, Dynamic Bayesian network

## Abstract

**Background:**

Healthy aging is a process of not only achieving good health but also increasing the life satisfaction of older adults aged 60 years and over, in which health behaviors play an important role. There is a lack of research on the time-varying dependencies between health, life satisfaction, and health behaviors, impeding a deeper understanding of healthy aging.

**Purpose:**

To develop an integrated framework for modeling the interrelationships among the components of healthy aging between multiple time slices.

**Methods:**

Based on the Chinese Longitudinal Healthy Living Survey (CLHLS) data in the three waves of 2011/2012, 2014, and 2017/2018, Bayesian network and dynamic Bayesian network are jointly employed to study the relationships among the components of healthy aging within one time slice, as well as to explore the time-varying dependencies among the components between time slices.

**Results:**

The results of structure learning reveal the direction of effects between different dimensions of health, with mental health and social health affecting physical health and self-rated health affecting both physical and mental health. In addition, health behaviors are found to affect mental health and social health, while self-rated health can influence life satisfaction. The parameters learned from the data show the magnitude and direction of concurrent effects, one-period lagged effects and two-period lagged effects between the factors, which find that the time-varying dependencies vary but are generally positive, long-term, and accumulative over time. In addition, the results of autoregressive effects show the positive predictive effects of health and life satisfaction.

**Conclusions:**

It confirms the influence pathway from health behaviors to multidimensional health to life satisfaction, and the time-varying dependencies among the components of healthy aging, which facilitates a deeper understanding of healthy aging. Combining the results of autoregressive effects and descriptive statistics, it further indicates that healthy aging is a comprehensive result arising from interactions of multiple factors. Policymakers should guide older adults aged 60 years and over to adopt healthier behaviors and ensure the long-term sustainability and continuity of policies.

**Supplementary Information:**

The online version contains supplementary material available at 10.1186/s12889-024-17752-2.

## Introduction

The population aged 60 years and older is expected to be double (2.1 billion) by 2050 [1], making population aging one of the major challenges facing most countries in the world, including China [2]. Promoting “healthy aging” is an important way for China to cope with this difficulty [3]. Healthy aging, proposed by the World Health Organization (WHO), is the process of developing and maintaining the functional ability that enables well-being in older adults [4], who are generally defined as adults aged 60 years and over in China [5–7]. Considering the controversies and complexity of defining and operationalizing healthy aging [8], it is of significance to explore the components of healthy aging and the relationship between the components for alleviating the problems caused by population aging and building a “Healthy China”.

Health is the key to the aging problem, and solving health problems can essentially resolve the negative impact of aging [3]. Considering health of older adults is not merely reflected in body condition, it is necessary to investigate the components of health from multiple dimensions and explore the relationships between them [3]. Healthy aging is not only reflected by the good health status of older adults, but more importantly, by the improvement of life satisfaction [9]. As health is a key determinant for older adults to realize their values, and achieve their well-being [10], the examination of the relationship between health and life satisfaction will constitute another crucial component of the research. To achieve the goal of healthy aging, that is, the well-being of older adults, many drivers should be considered, among which health behaviors are undoubtedly important and will have a profound impact on health outcomes [11–13]. Paying attention to research on the health behaviors of older adults aged 60 years and over, in addition to guiding them to adopt healthier lifestyle will be an important initiative in developing a healthy aging society.

Components of healthy aging, such as health and life satisfaction, are dynamic over time [14, 15] and influenced by various factors, such as the long-term impact of health behaviors on the health of older adults [16]. Hence, there may be time-varying characteristics of dependencies between variables, which is an important feature that needs to be examined [17]. To capture more fine-grained dynamic processes within and across health domains, studies with longer time series are necessary [18]. Similar to the different responses that economic decisions and economic variables (such as macroeconomic and financial) may make to short-, medium-, and long-term movements of uncertainty [19], research on the concurrent, one-period lagged, and two-period lagged dependencies among components of healthy aging may further deepen the understanding of healthy aging.

In health and aging domains, there are intricate correlations between health-related factors, that is, the state changes in one health-related factor can be both cause and effect of developments in other factors. Traditional regression models suffer from several limitations in dealing with such intricate relationships. First, regression models fail to incorporate seriously correlated variables in a single regression model and also fail to present the impact between dependent variable and dependent variable as well as the interaction between independent variable and independent variable, along with the reliance on specific assumptions and predefined relationships between dependent and independent variables [20], making it hard to accurately capture the full picture of healthy aging. In addition, it is also an aspect challenging for regression models to explore dynamic dependencies among multiple variables (including concurrent, short-term, and long-term effects) to identify the time-varying features of healthy aging. Bayesian network (BN) overcomes the limitations of regression models by identifying the interactions among multiple variables as well as detecting ignored important variables and relationships learned and trained from historical data without conventional specific assumptions and predefined relationships in regression models [20, 21]. Besides, it can represent complex causal structures with direct and indirect effects occurring on different levels in a straightforward and understandable graph [22]. As an extension of BN, dynamic Bayesian network (DBN) is capable of exploring time-varying dependencies between variables in different time slices [23, 24].

The contributes of this paper are three folds. First, Bayesian network and dynamic Bayesian network are introduced as the modeling methods in health and aging domains, which overcomes several limitations of regression models by obtaining more comprehensive and accurate conclusions. Second, an integrated framework is developed by simultaneously incorporating multidimensional health, life satisfaction, health behaviors of older adults aged 60 years and over as well as other drivers in a single dynamic model, contributing to capturing the whole picture of healthy aging. Finally, the time-varying dependencies among factors related to healthy aging are found, along with the autoregressive effects, which reveal the pathways to achieving healthy aging and shed further insights on the time-varying features of healthy aging. The findings provide empirical support for guiding older adults aged 60 years and over to adopt healthier behaviors and ensuring long-term sustainability and continuity of policies.

## Literature review

### Definition of healthy aging

In the current studies, there are still some controversies in the definition of healthy aging and the selected components. Almost no two studies define and operationalize healthy aging in the same way [8]. For example, Wagg et al. (2021) considered subjective well-being, physical health, self-rated health, biological aging, and cognitive function as components of healthy aging [8]. Wobbeking Sanchez et al. (2021) believed that the two key aspects of healthy aging are physical activity and life satisfaction [25]. Lara et al. (2013) selected the components of healthy aging from five domains, including (i) physiological and metabolic health, (ii) physical capability, (iii) cognitive function, (iv) psychological wellbeing, and (v) social wellbeing [26]. Combining WHO’s definition of healthy aging and previous studies, it may be appropriate to consider healthy aging as a process of not only achieving good health, but also increasing the life satisfaction of older adults aged 60 years and over. Thus, the components of healthy aging can be divided into two parts, the goals, namely health and life satisfaction, and the drivers.

### Association among different dimensions of health

#### The components of multidimensional health

Self-rated health is regarded as a useful indicator for understanding health issues, and it is an independent predictor of adverse health outcomes [27]. However, a single or a few indicators are not sufficient to reflect the whole picture of health status of older adults, leading to one-sidedness and uncertainty [3]. In 1946, in the preamble to its Constitution, the WHO gave a global definition of health: “a state of complete physical, mental and social well-being, and not merely the absence of disease” [28]. In recent years, there has been an increasing emphasis on the concept of overall health based on dimensions that include physical health, physical functioning, mental health, role function, and self-rated health [3]. Based on the above literature review, it is appropriate to consider self-rated health, physical health, mental health, and social health as the components of multidimensional health.

#### Relationship between multidimensional health

Self-rated health is a synthesis of various information available to a person that he or she understands belonging to “health” and has a clear biologic basis, thus self-rated health can be a sensitive barometer of physiologic states [29]. Besides, self-rated health could affect objective mental health, which can be explained by psychological mechanisms, such as experiencing health threats may threaten self-integrity and self-worth, possibly leading to negative emotional outcomes [30].

Mental health is a measure of mental status, consisting of positive affect and negative affect, which is also one of the components of subjective well-being (SWB) [31]. The relationship between physical health and SWB can be analyzed with reference to the top-down model [31], also known as the psychosomatic hypothesis [32]. The hypothesis suggests that the decline in physical health is usually a response to poor mental health [18]. There are several reasons why mental health can affect physical health. (i) Mental problems trigger related symptoms. (ii) Poor mental health status changes neurological, hormonal, and/or immune functions. (iii) Poor mental health status can lead to decreases in motivation and effort, indirectly affecting physical health.

The benefits of social health may depend on the group and the environment, in other words, being in social circles with the “right” people may be beneficial to the health outcomes [33]. One explanation for why social health affects physical and mental health is the benefits of social integration in that it gives meaning to a person’s life and thus reduces the chance of poor mental health or mortality [33]. Furthermore, three theoretical perspectives were proposed by Folland (2008) [34]. (i) Social capital reduces stress. (ii) Social capital provides information. (iii) Social capital brings responsibility to oneself and others.

### Relationship between health and life satisfaction

According to Maslow’s hierarchy of needs theory, health is a low-level and basic need for all individuals. When this low and basic need is not satisfied, it is difficult for older adults to pursue higher-level needs, leading to negative emotions [10]. This may be why health can affect quality of life/life satisfaction. There is also lots of literature to support this idea, for example, Zhang et al. (2021) found that self-rated health has a positive direct and indirect effect on quality of life/life satisfaction among Chinese older adults [10]. Chan and Wong (2022) confirmed the existence of causal effect of self-rated health on life satisfaction [35].

### Influence of health behaviors on health

To improve the health and life satisfaction of older adults aged 60 years and over, many factors should be considered, among which lifestyle is extremely important. According to the knowledge of social epidemiology, lifestyle can be effective in preventing diseases and mental problems [36]. The WHO (1998) stated that “a healthy lifestyle is a way of living that lowers the risk of being seriously ill or dying early” [37]. There is no unified academic definition of healthy lifestyles or what healthy lifestyles formally means. Peel et al. (2005) summarizes the behavioral determinants of healthy aging, identifying several behavioral factors including physical activity, body mass index, diet, alcohol use, smoking status, and health practices [38]. Fernández-Ballesteros et al. (2022) identified four healthy lifestyle factors: regular exercises, a healthy diet, sufficient sleep, and weight control [37]. Besides, more diverse perspectives exist, with some studies regarding lifestyle factors as smoking, drinking, eating diets, and physical inactivity [39], others considering lifestyle as exercises, diets and intellectual activity [40]. Although there are still controversial views about the operational definition of lifestyles, as Clegg et al. (2013) suggests, “there is an emerging evidence that appropriate exercise and nutrition can stabilize frailty and thus reduce the resulting vulnerability” [41, 42], healthy diets (especially intake of fruits and vegetables) and physical exercise are repeatedly considered to be the lifestyle factors with the influence on health confirmed as protective in research studies [43, 44]. For example, Brach et al. (2004) found that older adults who usually participate in moderate-intensity exercise had better physical function [11]. The high frequency of vegetable and fruit intake is negatively associated with all-cause mortality of older adults [13]. Not merely improving physical health, Hodge et al. (2013) found that a healthy diet could reduce the risk of depression and anxiety [45]. Moreover, Bots et al. (2008) found that physical activity and moderate alcohol consumption might protect against depression in old age [46]. The paper focuses on the health-supporting behaviors or health protective behaviors, and thus employ healthy diets and physical exercise as variables of interest, which are called “health behaviors”.

### Research framework

**Fig. 1:**
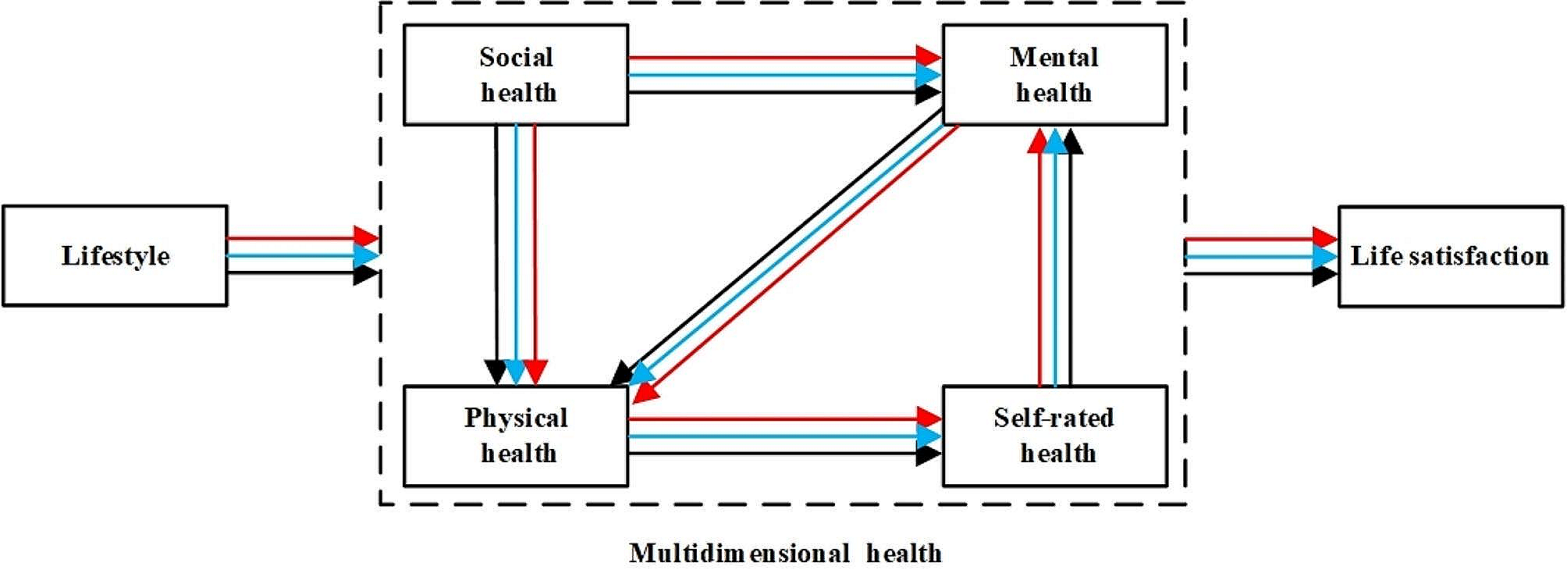
Research Framework *Note*: The black lines represent the effects of the variables on other variables in the same period (concurrent effects). The blue lines represent the effects of the variables in the preceding one period on other variables (one-period lagged effects), and the red lines represent the two-period lagged effects. The colored lines in the following figures represent the same meanings

Based on the literature review and the purpose of this article, the research framework is presented in Fig. 1.

## Data and methods

### Data

As the first national-wide and longitudinal survey on the health of elderly Chinese, the Chinese Longitudinal Healthy Longevity Survey (CLHLS) provides the original data source^1^ [47]. CLHLS is a collaborative project between Duke University and Peking University, aiming to shed further light on the determinants of healthy longevity of human beings [48]. The CLHLS has the world’s largest sample size of the oldest old aged 80 + with comparable samples of the younger old with rich data information, which has provided great opportunities and potentials for scientific research and policy analyses [49]. To provide representative and meaningful information, the CLHLS employs internationally compatible questionnaires [47], which covers many research contents related to the family socio-economic status, daily living conditions, life satisfaction and health status of older adults [50]. Its baseline survey began in 1998, and seven follow-up survey waves were conducted in 2000, 2002, 2005, 2008–2009, 2011–2012, 2014, and 2017–2018 [51]. The details of sampling design are as follows [49, 52].

(1) The survey was based on a randomly selected sample of elderly Chinese from a half of the counties/cities in 23 Han-ethnicity-dominant provinces in China (Eight provinces in northwest China were excluded from the CLHLS due to the unreliable information on self-reported age), with these areas covering 85% of the total Chinese population [53].

(2) Given the numerous health issues and care needs of the oldest old group [66], it calls for more attention to this group. However, information pertaining to the oldest old group was very limited because the sample size of this group in other social surveys was relatively small, and any sample selection that was proportionate based on the actual age structure of the population would be highly concentrated towards relatively younger elderly people and female elderly. To ensure adequate sample size for the oldest old especially the male oldest old sub-groups and to eliminate problems often encountered by proportionate sampling, such as difficult to analyze determinants of health and gender differences among oldest old because of small sample size, CLHLS did not follow the procedure of proportional sampling design, but instead interviewed nearly all centenarians and over-sampled the oldest-old of more advanced ages, especially among males [54, 55]. Specifically, all centenarians living in the selected sites who voluntarily participate in the survey were visited, and for each centenarian interviewed with the last digit of identification number 0–4, one 80–84 year-old, one 90–94 year-old, one 70–74 year-old resident nearby were interviewed. For each centenarian interviewed with the last digit of identification number 0–4, one 85–89 year-old, one 95–99 year-old, one 75–79 year-old, and one 65–69 year-old resident nearby were interviewed. The gender of interviewees age 65–99 was defined by birth date of the centenarian interviewed. Male older adult was interviewed if birth month of the centenarian was between January and June, and female older adult was interviewed if the birth month of the centenarian was between July and December. The basic idea of selecting the sample in this way was to ensure that the number of centenarians and the number of older persons in each age group were roughly equal. While the number of male and female interviewees are also roughly the same by single age. The validity of the targeted random sampling is tested and accepted by scholars who use seven waves of CLHLS datasets [49, 52].

(3) In the 1998 and 2000 waves, the CLHLS focused only on older adults aged 80 and over. In order to obtain a comparable sample, it additionally incorporated younger older adults aged 65–79 since 2002, broadening the minimum cut-off age for respondents to 65 years. Additional 4478 adult children aged 35–65 of older interviewees were interviewed in 2002 and 2005 wave in eight coastal provinces (Guangxi, Guangdong, Fujian, Jiangsu, Zhejiang, Shandong, Beijing, and Shanghai) [56]. Seven longevity areas were included in the CLHLS after 2009, where adult children aged 60 and over were interviewed. To maintain a large enough sample size, CLHLS replaced the deceased respondents by recruiting new participants with in the same gender and roughly the same age as them in the 2000, 2002, 2005, and 2008–2009 follow-up waves. However, in the 6th survey in 2011–2012, only follow-up interviews without new participants were conducted, except for the longevity areas where new participants were still recruited [54]. Considering that all respondents aged under 65 years interviewed before 2011 had died or lost to follow-up [57], the eligible respondents were aged 60 and over in the sixth wave of CLHLS data.

(4) The data were collected through in-home interviews by trained interviewers who are local staff members from the county-level network system of the National Bureau of Statistics of China. All interviewers have received over 12 years of schooling, and most have earned a college degree. Each interviewer was accompanied by a local doctor, a nurse, or a medical college student so that some health check-ups could be performed. The Research Ethics Committees of Peking University and Duke University (IRB00001052–13,074) granted approval for the Protection of Human Subjects for the Chinese Longitudinal Healthy Longevity Survey, including collection of the data used for present study. The survey respondents gave informed consent before participation [2].

**Fig. 2:**
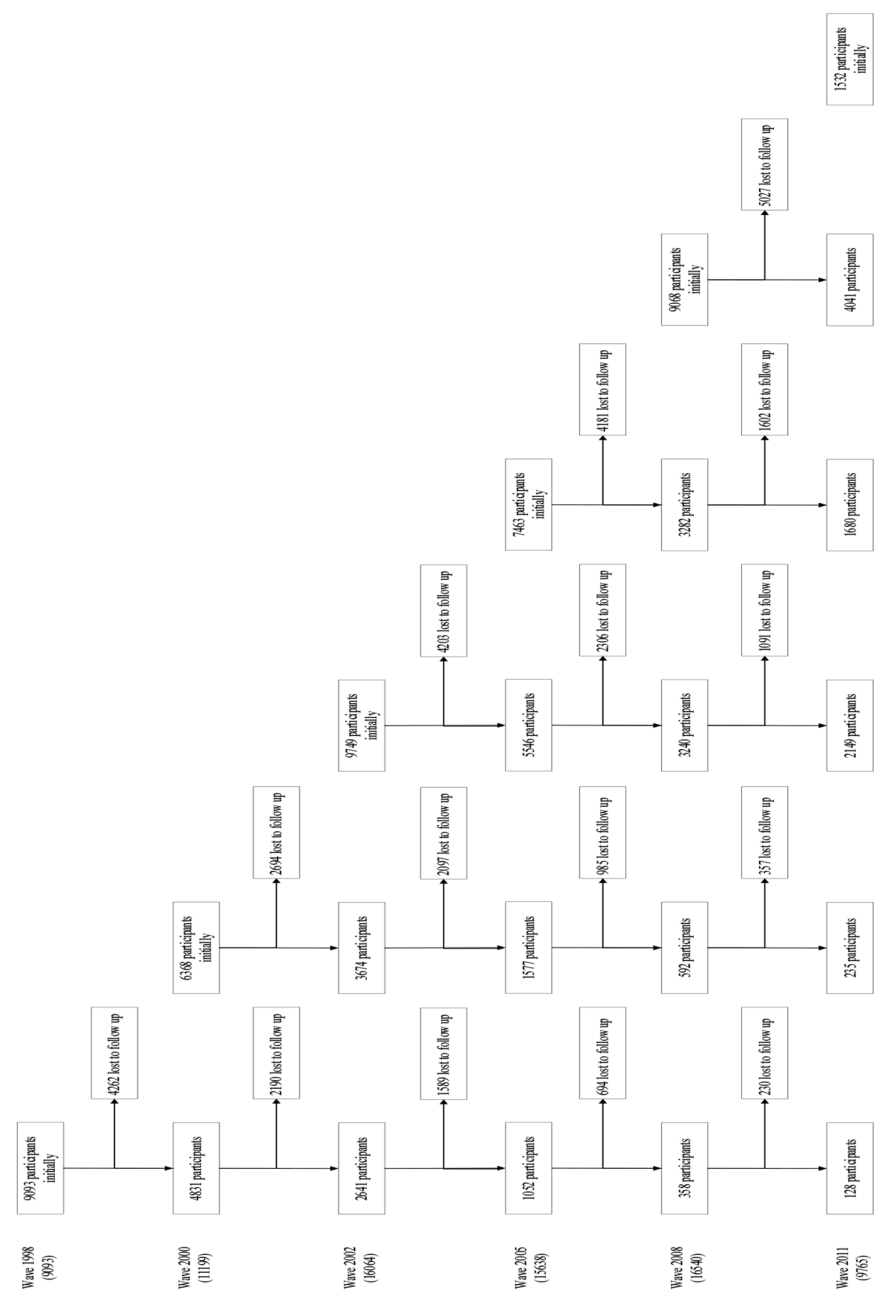
Flowchart of CLHLS cohort from wave 1998 to 2011 *Note*: Due to restrictions on size, the figure is rotated 90 degrees counterclockwise. The leftmost column is the cohort of respondents interviewed in the first wave. Each added column is a new cohort of respondents, joining the original respondents to constitute the full sample for the wave

The data quality have been described elsewhere [58]. Considering the purpose of this paper to investigate time-varying dependencies (including concurrent effects, one-period lagged effects, and two-period lagged effects), data from three periods are needed. Therefore, the sixth wave of CLHLS data (incorporates information in 2011–2012, 2014, and 2017–2018) were employed in this paper. A total of 9,765 respondents are employed as our initial sample. More details of the CLHLS sample structure can be found in Fig. 2, which presents the flowchart of CLHLS cohort from wave 1998 to 2011.

**Fig. 3:**
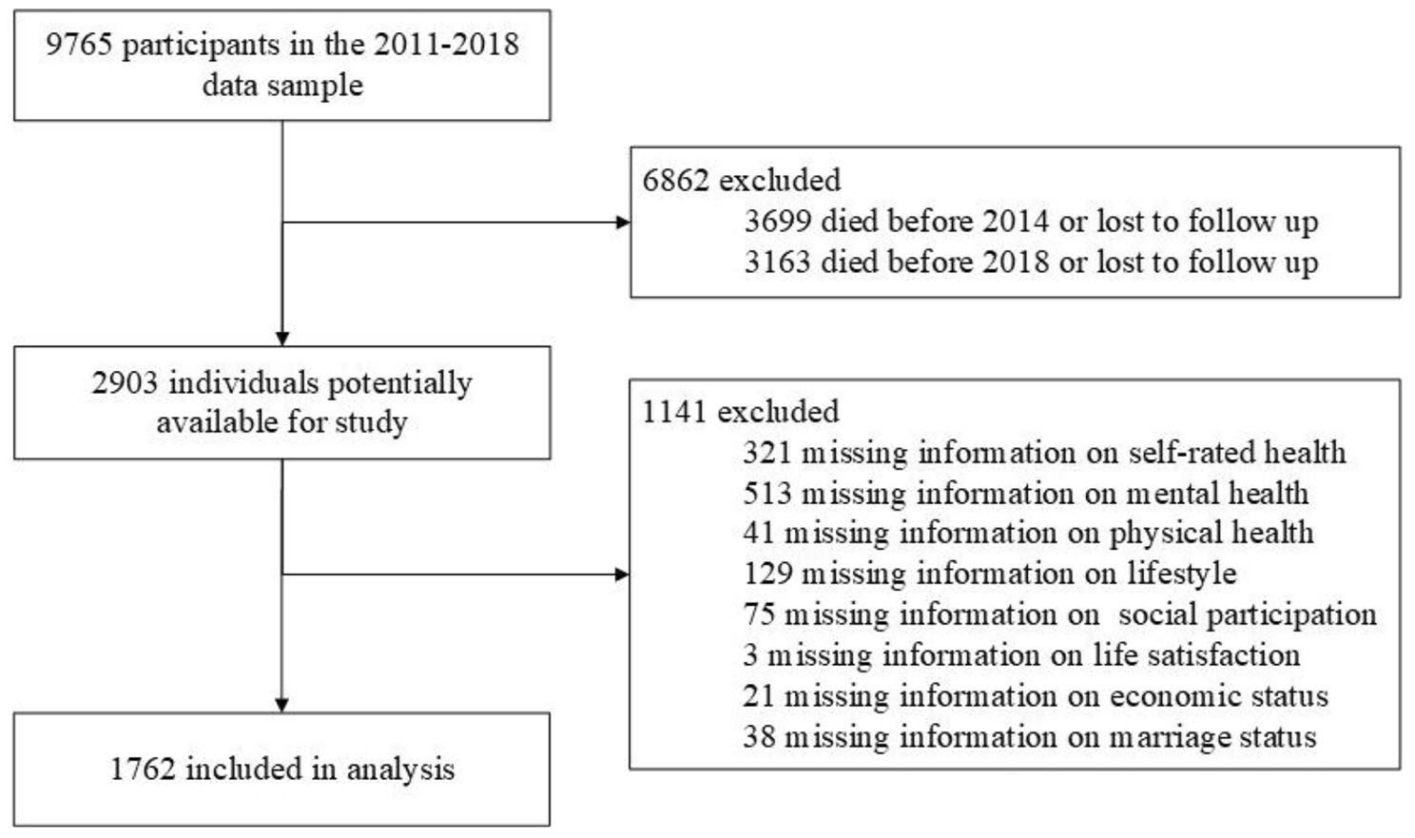
Data preprocessing procedure

3699 and 3163 samples were respectively dropped due to death or loss to follow-up in the 2014 and 2018 waves. In addition, considering missing data on the concerned variables, we removed 1,141 samples, resulting in a total of 1,762 samples as the final analytic sample. All respondents are older adults aged 60 years and over. Data preprocessing procedure is shown in the following figure (Fig. 3).

### Variables

Considering that the objective of this paper is to explore the pathways to achieving healthy aging, that is, to investigate what drivers and what influence paths lead to better health-related outcomes, which gives motivation for dichotomizing the variables into relatively good and relatively bad. Besides, For the clarity and brevity of representation in BN and DBN, dichotomous measures are recommended. The dependencies between variables are captured by the linkage between them and by the changes in conditional probabilities of the linked variables being high-level state given certain state of one variable, which gives dichotomous measures a good fit in enabling more understandable conclusions, whereas multi-state measures may confuse conclusions. In addition, changes in the states of a health-related outcome caused by other factors ultimately boil down to a dichotomous conclusion that whether these drivers render the outcome better or worse. Therefore, the variables of interest are dichotomized, that is, each variable can be regarded as whether the event “good outcome” occurs or not, and the associations between different events are modeled by BN and DBN to capture the dependencies between the variables [23, 24]. The norm was not imposed on the covariates.

**Self-rated health** was measured by a single question in which respondents were asked to rate their overall health status. For the sake of model estimation as well as ease of results interpretation, the “poor” and “very poor” answers were grouped into “poor” (coded as 0), and the “very good”, “good” and “fair” answers were grouped into “good” (coded as 1) [58–60].

**Mental health** was measured by three negative questions and four positive questions in the survey data. All questions had five responses, where “always”, “often” and “sometimes” were defined as “occurrence of negative events” for negative questions, while “seldom” and “never” as “occurrence of negative events” for positive questions. The presence of any negative events in the seven questions was defined as a poor level of mental health (coded as 0), and otherwise a high level of mental health (coded as 1) [61].

**Physical health** was measured by an 8-item instrumental activities of daily living (IADL) disability scale. The respondent was defined as elderly with IADL disability (coded as 0, otherwise 1) if he/she answered unable to complete any of the 8-item IADL disability [62].

**Life satisfaction** was measured by the question “How do I feel about my quality of life?”. In line with previous research, the “very high”, “high” and “fair” answers were grouped into “high” (coded as 1), while “low” and “very low” answers were grouped into “low” (coded as 0) [47].

**Social health** was measured by the degree of social participation, which was measured with reference to Jiehua et al., (2017). The respondents were considered as socially healthy (coded as 1) if they engage in group leisure-time activities (such as playing cards or mahjong) at least once a week, participate in social organization activities at least once a month, or traveled at least once in the last two years. Otherwise, the respondent was considered as not socially healthy (coded as 0) [63].

**Health behaviors** of the respondent were measured by three factors: daily intake of fruits, daily intake of vegetables, and regular physical activity performance [13, 64]. The respondent was defined as having health behaviors (coded as 1) if he/she answered consuming fruits and vegetables almost every day or quite often, along with answering doing exercise regularly. Otherwise, the respondent was defined as not having health behaviors (coded as 0).

**Covariates** included several socio-economic variables. There are significant gender differences in the health of older adults, thus gender was included in the framework of the study, where males were coded as 1 and females were coded as 0. According to WHO [65], older adults aged 60 years and over are called the Elderly and those aged over 80 and over are defined as the Oldest-old, which is consistent with China [5, 7, 52]. Here, older adults who are the elderly but not the oldest old are named the younger old (aged 60–79 years, coded 0). Considering that the oldest old suffer particularly pronounced decline in physical health and other key areas of life [66], achieving healthy ageing calls for additional attention to them. Thus, older adults aged 80 and over are further categorized as octogenarians (aged 80–89, coded 1), nonagenarians (aged 90–99, coded 2), and centenarians (aged 100 and over, coded 3). In addition, residential type (urban = 1, town or rural = 0) and marital status (married = 1, or unmarried = 0) are also generally recognized as important covariates [2, 16]. The economic status is associated with quality of life and access to healthcare, which was assessed using self-reported economic condition compared to other locals, with response options “very rich” and “wealthy” coded 3, “average” coded 2, “poor” and “very poor” coded as 1 [47].

### Methods

#### Model specification

Bayesian network (BN) modeling was employed as the empirical methodology in this paper. BNs, also known as probabilistic graphical models or belief network models, combine probability theory and graph theory. A BN model consists of two parts: a directed acyclic graph and a conditional probability table. Directed acyclic graphs express the complex dependencies between variables (which variables affect which other ones) while conditional probability tables show the strength of the dependency between variables. For a set of random variables ${\text{X}}_{1}, {\text{X}}_{2}, \dots, {\text{X}}_{\text{n}}$, the joint distribution is:

1 $$ {P\left( {{{\rm{X}}_1},{{\rm{X}}_2}, \ldots,{{\rm{X}}_{\rm{n}}}} \right) = \prod\limits_{{\rm{i}} = 1}^{\rm{n}} {\rm{P}} \left( {{{\rm{X}}_{\rm{i}}}|{\rm{Pa}}\left( {{{\rm{X}}_{\rm{i}}}} \right)} \right)}$$

where ${\text{X}}_{\text{i}}$ is the $\text{i}$th node in the directed acyclic graph, $\text{P}\text{a}\left({\text{X}}_{\text{i}}\right)$ are the parents of node ${\text{X}}_{\text{i}}$, $\text{P}\left({\text{X}}_{\text{i}}\right|\text{P}\text{a}\left({\text{X}}_{\text{i}}\right))$ is the conditional probability of node ${\text{X}}_{\text{i}}$ given $\text{P}\text{a}\left({\text{X}}_{\text{i}}\right)$.

The dynamic Bayesian network (DBN) model is applied to capture the dynamic dependencies of variables at different time slices. A DBN is defined by ${\text{B}}_{0}$ and ${\text{B}}_{\to }$, where ${\text{B}}_{0}$ is a standard BN model that represents the unconditional initial state distribution, and ${\text{B}}_{\to }$ is a two-slice temporal BN which defines the transition of conditional probability distributions of variables between time $\text{t}-1$ and $\text{t}$:

2 $$ \begin{array}{*{20}{c}}{P\left( {{{\rm{X}}_{\rm{t}}}|{{\rm{X}}_{{\rm{t}} - 1}}} \right) = \prod\limits_{{\rm{i}} = 1}^{\rm{n}} {\rm{P}} \left( {{\rm{X}}_{\rm{t}}^{\rm{i}}|{\rm{Pa}}\left( {{\rm{X}}_{\rm{t}}^{\rm{i}}} \right)} \right)}\end{array}$$

where ${\text{X}}_{\text{t}}^{\text{i}}$ is the $\text{i}$th node at time t, and $\text{P}\text{a}\left({\text{X}}_{\text{t}}^{\text{i}}\right)$ are the parents of ${\text{X}}_{\text{t}}^{\text{i}}$. The joint probability distribution of a set of random variables for a sequence of length $\text{T}$ is:

3 $$ \begin{array}{*{20}{c}}{P\left( {{{\rm{X}}_1},{{\rm{X}}_2}, \ldots,{{\rm{X}}_{\rm{T}}}} \right) = {{\rm{P}}_{{{\rm{B}}_0}}}\left( {{{\rm{X}}_1}} \right)\prod\limits_{{\rm{t}} = 1}^{\rm{T}} {{{\rm{P}}_{{{\rm{B}}_ \to }}}} \left( {{{\rm{X}}_{\rm{t}}}|{{\rm{X}}_{{\rm{t}} - 1}}} \right)}\end{array}$$

In a DBN model, the change process of conditional probability of random variables is a Markov process, meaning that each variable is directly influenced only by the previous variable. The change process of conditional probability of adjacent time is stable, and the graph structures at each time slice are the same.

The key of DBNs is learning, which includes two parts: structure learning and parameter learning.

#### Structure learning

Structure learning is to learn BN structure from a given data set. Network structures can be constructed using expert knowledge (priori knowledge) or available observations, which were combined in our research to complement and constrain each other in an attempt to find a realistic network structure [23, 67]. Therefore, a semi-structure learning process was adopted in this study, which could exceed merely unsupervised learning methods.

Specifically, the initial network was learned from the data at the initial time using algorithms [68]. Peter-Clark (PC) algorithm was applied in this paper to learn the structure within one time-slice since it was capable to recover a graph the same as the realistic model by identifying causal relationships between variables [69]. The level of significance was set as the default value 0.05 for the learning process [70]. In addition, some constraints based on the priori knowledge were set to simplify structure learning and deny some unreasonable relationships, which would not affect the validity of the analysis [23]. The details were as follows.

(1) Socio-economic variables, such as age, gender, residential type, marital status, and economic status were not allowed to be influenced by health status, health behaviors, and life satisfaction.

(2) Connections among socio-economic variables were impossible, such as no linkage between age, gender, residential type, marital status, and economic status.

The structure between time slices (i.e., the network structure of the transfer network) was constructed manually from expert knowledge based on literature [23, 24, 67], as follows.

(1) Self-rated health and social health was assumed to both have one- and two- period lagged effects on mental health and physical health [30, 71–73]. Mental health was assumed to be a predictor of future physical [18].

(2) Health behaviors were assumed to have a one- and two-period lagged effect on mental and physical health [74, 75].

(3) As a determinant of life satisfaction, self-rated health was assumed to have lagged effects on it [76].

(4) It was assumed that there were autoregressive effects for different dimensions of health as well as life satisfaction [35, 73, 77].

#### Parameter learning

Given this semi-learned network, according to the advice of Guo et al. (2020), the EM (expectation maximization) algorithm can be used to learn the parameters [24]. Based on the learning results, when a node of interest is provided with evidence, the probabilities of a node linked to it (observed node) will be updated, and the relative probability differences before and after the update can be used to analyze the dependencies between the nodes [23, 24]. Assume the initial probability of a certain state of a variable is ${\text{p}}_{0}$, and the updated probability is ${\text{p}}^{\text{*}}$. The relative probability difference ${\text{p}}_{\text{d}\text{i}\text{f}\text{f}}$ is calculated as follows.

4 $$ \begin{array}{*{20}{c}}{{{\rm{p}}_{{\rm{diff}}}} = \frac{{{{\rm{p}}^{\rm{*}}} - {{\rm{p}}_0}}}{{{{\rm{p}}_0}}}}\end{array}$$

For ease of understanding, the high-level state of each node was concerned in our paper. For instance, the change in the probability of good physical health given the evidence of good mental health was examined. In addition, for the convenience of analysis, this paper set period $\text{t}2$ as the base period $\text{t}$. Thus, given evidence for nodes of interest at period t2, t1, and t0, the relative probability differences of observed nodes at period t2 could respectively capture concurrent effects, one-period lagged effects and two-period lagged effects.

## Analysis and results

### Descriptive statistics

A total of 1,762 participants were employed as the analytic sample, and the descriptive statistical information is shown in Table 1. The proportions of males and females are similar. The majority of the samples are the younger old or octogenarians, mostly living in towns or rural areas. In addition, the economic status of the elderly is mostly self-rated as medium. The difference between the number of the married and unmarried elderly is not significant. Statistics show that the life satisfaction and self-rated health of older adults aged 60 years and over are relatively high, while mental health and social health as well as health behaviors are poor, and the physical health of the elderly is relatively fair. As time advances, life satisfaction of older adults gradually increases, with insignificant changes in self-rated health and a gradual deterioration in all other health indicators and health behaviors. Descriptive statistics grouped by gender are presented in Appendix 2, where several differences in various variables between males and females can be found.

**Table 1:** Descriptive statistics of samples

Variable	Category	All		Year = 2011		Year = 2014		Year = 2018	
		N	P	N	P	N	P	N	P
Sex	0	2598	49.15%	866	49.15%	866	49.15%	866	49.15%
	1	2688	50.85%	896	50.85%	896	50.85%	896	50.85%
City	0	4490	84.94%	1505	85.41%	1506	85.47%	1479	83.94%
	1	796	15.06%	257	14.59%	256	14.53%	283	16.06%
Age	1	2953	55.86%	1238	70.26%	1051	59.65%	664	37.68%
	2	1703	32.22%	414	23.50%	537	30.48%	752	42.68%
	3	515	9.74%	83	4.71%	139	7.89%	293	16.63%
	4	115	2.18%	27	1.53%	35	1.99%	53	3.01%
Satisfaction	0	180	3.41%	88	4.99%	44	2.50%	48	2.72%
	1	5106	96.59%	1674	95.01%	1718	97.50%	1714	97.28%
Self-rated health	0	665	12.58%	214	12.15%	226	12.83%	225	12.77%
	1	4621	87.42%	1548	87.85%	1536	87.17%	1537	87.23%
Mental health	0	3798	71.85%	1210	68.67%	1255	71.23%	1333	75.65%
	1	1488	28.15%	552	31.33%	507	28.77%	429	24.35%
Health behaviors	0	4286	81.08%	1403	79.63%	1435	81.44%	1448	82.18%
	1	1000	18.92%	359	20.37%	327	18.56%	314	17.82%
Social health	0	3744	70.83%	1191	67.59%	1223	69.41%	1330	75.48%
	1	1542	29.17%	571	32.41%	539	30.59%	432	24.52%
Physical health	0	2333	44.14%	593	33.65%	694	39.39%	1046	59.36%
	1	2953	55.86%	1169	66.35%	1068	60.61%	716	40.64%
Economic status	1	583	11.03%	246	13.96%	164	9.31%	173	9.82%
	2	3647	68.99%	1185	67.25%	1277	72.47%	1185	67.25%
	3	1056	19.98%	331	18.79%	321	18.22%	404	22.93%
Marriage	0	2376	44.95%	697	39.56%	761	43.19%	918	52.10%
	1	2910	55.05%	1065	60.44%	1001	56.81%	844	47.90%

Note: N is the number of samples, while P is the proportion of the samples to the total samples

### Results of structure learning

**Fig. 4:**
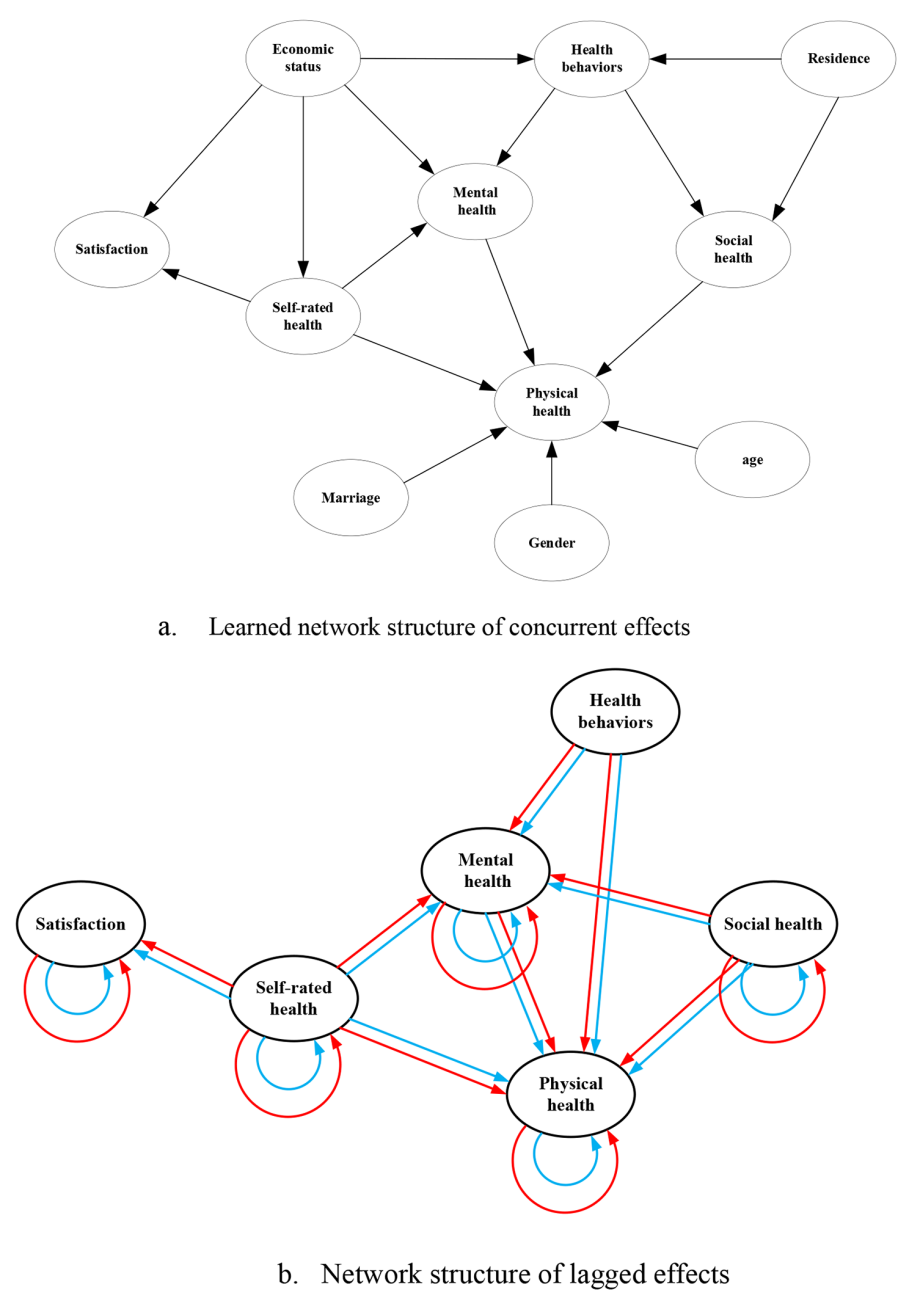
The network structures *Note*: Figure (**a**) describes the initial Bayesian network structure, and Figure (**b**) describes the transfer network between time slices, both of which are single-column fitting images

GeNIe software^2^, a professional Bayesian network visualization software, was applied to model the BN to explore the relationship between variables. Results of structure learning within one time slice are shown in Fig. 4 (a).

From the learned network structure, causal dependencies among the different dimensions of health are found. Specifically, self-rated health affects mental health and physical health, while mental health and social health have effects on physical health. Health behaviors directly link to mental health, and interestingly, the influence of health behaviors on social health is observed, suggesting that older adults aged 60 years and over with different health behaviors may exhibit varying levels of social engagement. In addition, the effect of self-rated health on life satisfaction is observed. Socio-demographic variables, such as age, gender, and marriage are directly linked with physical health, whilst the links from residential type to social health and health behaviors are also observed. Moreover, economic status is found to affect health behaviors, mental health, self-rated health, and life satisfaction status.

In the unrolling structure of the DBN model, the same network was copied to each time slice, and the temporal dependencies among the nodes were constructed based on numerous literatures, which are shown in Fig. 4 (b).

### Results of parameter learning

Based on the constructed network, the parameters of the entire network were obtained using the EM algorithm. Given the evidence for nodes of interest, the relative probability changes of observed nodes can be used to capture time-varying dependencies between components of healthy aging, which are shown in the following sections.

#### Changes in health, life satisfaction, and health behaviors given socio-demographic status

Taking demographic variables as the parent nodes, both the updated probability and the relative probability differences (shown in parentheses) of physical health are presented in Table 2. The oldest old are in poorer physical health than the younger old, which is a natural consequence of the aging process. However, the likelihood of maintaining good physical health gradually increases as the age category increases among the oldest old, possibly due to the heightened body awareness among the older adults at an advanced age. Gender plays a role in the physical health, which highlights the “gender paradox” in health and mortality, where women generally live longer than men but tend to have worse health during survival [2]. Moreover, married older adults aged 60 years and over display better physical health compared to unmarried older adults, reflecting a phenomenon known in previous studies as the “protective effect” of marriage [78].

**Table 2:** Differences in physical health for different age, gender, and marital categories

Socio-demographic status		Physical health(t)
	No change	48.32%
Age	60–79	53.45% (10.60%)
	80–89	45.04% (-6.80%)
	90–99	45.25% (-6.36%)
	>=100	47.78% (-1.13%)
Gender	Woman	45.74% (-5.34%)
	Man	50.82% (5.16%)
Marriage	No	46.85% (-3.06%)
	Yes	49.93% (3.33%)

As shown in Table 3, health behaviors of older adults aged 60 years and over are observed to have a significant urban-rural difference, specifically, the probability of urban older adults adopting health behaviors increases by 124.40%. This urban-rural difference also exists in social health of older adults. These findings may be attributed to the better infrastructure, the wider spread of knowledge, and the more proactive atmosphere in urban areas, which led to more prevalence of healthy behaviors and social participation.

**Table 3:** Differences in health behaviors and social health for different residential types

Socio-demographic status		Health behaviors(t)	Social health(t)
	No change	17.89%	24.04%
Residence	Rural	13.63% (-23.84%)	23.18% (-3.56%)
	Urban	40.15% (124.40%)	28.51% (18.58%)

As shown in Table 4, it’s found that respondents with lower level of economic status exhibits poorer mental health, self-rated health, and life satisfaction, along with a lower likelihood of having healthier behaviors. The high level of self-rated economic status is generally associated with contrasting outcomes [39, 79], but paradoxically, high economic status older adults are less satisfied with their lives compared to middle economic status older adults. These results may reveal a phenomenon that the mental state and well-being of older people, as well as the health behaviors they adopt, are related to their material foundations and living conditions.

**Table 4:** Differences in mental health, life satisfaction, self-rated health, and health behaviors for different economic status

Socio-demographic status	Self-rated health(t)	Mental health(t)	Satisfaction(t)	Health behaviors(t)
No change	87.31%	29.98%	96.05%	17.89%
Low	69.07% (-20.88%)	25.96% (-13.39%)	84.36% (-12.17%)	8.56% (-52.17%)
Medium	88.22% (1.04%)	26.23% (-12.52%)	97.70% (1.72%)	15.74% (-12.00%)
High	92.47% (5.91%)	42.74% (42.58%)	96.21% (0.16%)	28.22% (57.73%)

#### Changes in health and life satisfaction given status of multidimensional health

In Table 5 and the following tables, “t” indicates the period in which the observed node is situated, while “t-1” and “t-2” indicate one period and two periods ahead of it. Table 5 shows that older adults aged 60 years and over with good mental health have an increased probability of good physical health (7.06%). Furthermore, both positive one-period lagged effect (4.01%) and two-period lagged effect (5.59%) are observed. The concurrent effect is larger than lagged effects, possibly due to mental problems leading to symptoms like insomnia and emotional changes altering the levels of biological factors like hormones [18], all of which will have a greater impact on current physical health than future physical health. The two-period lagged effect is stronger than the one-period lagged effect, highlighting the positive and profound impact of mental health on physical functioning in older adults aged 60 years and over, which may be long-term and may accumulate over time.

**Table 5:** Time-varying effects of mental health on physical health

Health		Physical health(t)
	No change	48.32%
Mental health	t	51.74% (7.06%)
	t-1	50.26% (4.01%)
	t-2	51.03% (5.59%)

As shown in Table 6, the time-varying effects of self-rated health on physical health are all positive but close to 0. It indicates although subjective health may be a predictor of physical health outcomes [29, 32, 75], it is not a significant driver. The positive concurrent effects of self-rated health on both mental health and life satisfaction can be found [30, 35], indicating that good self-assessment of health may provide older adults aged 60 years and over with positive mental states and emotions. In terms of lagged effects, the one-period lagged effect of self-rated health on mental health is found to be insignificant, while the two-period lagged effect is significantly positive. In addition, the two-period lagged effect and one-period lagged effect of self-rated health on life satisfaction are both positive and similar in magnitude. The results suggest that self-rated health will improve future mental health, but it takes some time to manifest. Besides, self-rated health has a long-term effect on life satisfaction, which may accumulate over time.

**Table 6:** Time-varying effects of self-rated health on physical health, mental health, and life satisfaction

Health		Physical health (t)	Mental health (t)	Satisfaction (t)
	No change	48.32%	29.98%	96.05%
Self-rated health	t	48.40% (0.15%)	30.38% (1.33%)	97.54% (1.55%)
	t-1	48.41% (0.19%)	29.97% (-0.03%)	96.69% (0.67%)
	t-2	48.39% (0.13%)	30.26% (0.93%)	96.81% (0.80%)

As shown in Table 7, social health has a positive concurrent effect on physical health (8.26%), in addition, one-period lagged (6.35%) and two-period lagged effects (7.01%) of social health on physical health are also observed, suggesting that social health has a long-term positive influence on physical health that will accumulate over time. Plausible explanations include the persistent impact caused by the provision of health-related information and the cultivation of health awareness through social networks. Concurrent effect of social health on mental health is not found, possibly since social participation may improve mental health while also bringing about psychological distress [80]. The one-period lagged effect (12.89%) and two-period lagged effect (8.70%) of social health on mental health are positive but diminishing in order, indicating that benefits such as emotional support provided by social relationships [73] tends to dissipate over time.

**Table 7:** Time-varying effects of social health on physical health and mental health

Health		Physical health (t)	Mental health (t)
	No change	48.32%	29.98%
Social health	t	52.31% (8.26%)	
	t-1	51.39% (6.35%)	33.84% (12.89%)
	t-2	51.71% (7.01%)	32.59% (8.70%)

#### Changes in multidimensional health given health behaviors

As shown in Table 8, health behaviors have a significant positive concurrent effect (39.87%) on mental health, while no significant concurrent effect of health behaviors on physical health is observed. It may be due to the fact that changes in health behaviors can affect mental health, but it takes some time before it affects physical status. The two-period lagged effects of health behaviors on both mental health and physical health are all positive and stronger than one-period lagged effects, which supports this view that contributions of health behaviors to health status are long-term and will accumulate over time. Besides, it also reveals that older adults aged 60 years and over with healthier behaviors are more likely to have greater social health. A possible reason is that older adults with healthier behaviors are more health-conscious and are more likely to participate in a “circle” of health-conscious people [33], thus adopting more positive attitudes toward life, and being more inclined to participate in social activities and make friends.

**Table 8:** Time-varying effects of health behaviors on physical, mental health, and social health

Health behaviors		Physical health (t)	Mental health (t)	Social health (t)
	No change	48.32%	29.98%	24.04%
Health behaviors	t		41.93% (39.87%)	34.67% (44.20%)
	t-1	49.23% (1.88%)	38.87% (29.65%)	
	t-2	50.11% (3.69%)	39.72% (32.50%)	

#### Autoregressive effects

To capture the autoregressive effects, Table 9 shows the results of updated conditional probability and relative probability differences of multidimensional health and life satisfaction given evidence for themselves at previous time. Positive one-period autoregressive and two-period autoregressive effects indicate the positive predictive capabilities over time.

**Table 9:** Autoregressive effects of multidimensional health and life satisfaction

Autoregressive effects		Self-rated health (t)	Physical health (t)	Mental health (t)	Social health (t)	Satisfaction (t)
	no change	96.05%	48.32%	29.98%	24.04%	96.05%
Self-rated health	t-1	89.41% (2.41%)				
	t-2	88.77% (1.68%)				
Physical health	t-1		51.45% (6.46%)			
	t-2		50.59% (4.69%)			
Mental health	t-1			36.45% (21.57%)		
	t-2			35.65% (18.93%)		
Social health	t-1				43.83% (82.34%)	
	t-2				40.61% (68.91%)	
Satisfaction	t-1					96.65% (0.62%)
	t-2					96.75% (0.73%)

### Sensitivity analysis

The paper conducts a sensitivity analysis to examine the robustness of conclusions, which includes the sensitivity analysis for data preprocessing procedure and sensitivity analysis for assumptions of time-varying dependencies.

#### Sensitivity analysis for data preprocessing procedure

Considering the exclusion of 1141 samples due to the presence of missing value, some efforts are made to prevent inconsistent estimates of the relationships between the variables. There are 837 samples lacking key information about mental health, self-rated health, and life satisfaction that are additionally marked in the questionnaires to be answered by participants in person. The key information is highly heterogeneous to individuals so that samples with missing key information are suggested to be dropped. In addition, as this paper aims to explore pathways to healthy aging, these samples, who were unable to answer key questions in questionnaires by themselves, may not be representative of the population. For the other 304 samples, a significance test for the difference between analytic sample (including 1762 samples) and the sample without missing key information (including 2066 samples) is conducted. The results of significance test are shown in Appendix 1, which suggest that there is no statistically significant difference.

In addition, multiple imputation of missing values is conducted based on Random Forests. Based on the imputed 2903 samples, the structure learning results of Bayesian network are shown in Appendix 1. In addition, transfer network (shown in Figs. 2 and 3 in Appendix 1) is also built to develop the dynamic Bayesian networks, followed by obtaining the results of parameter learning. Compared to the original conclusions, there are only two paths have changed (dependency between physical health and mental health and dependency between physical health and social health), which are also shown in Appendix 1. Besides, the overall pathway from health behaviors to multidimensional health to life satisfaction is not changed. The time-varying dependencies among the variables are generally similar. Considering the potential errors of multiple imputation on the key information, along with the fact that there are already both valid perspectives in the academia explaining the relationship between physical and mental health, e.g., the top-down model and bottom-up model, the conclusions are relatively robust.

#### Sensitivity analysis for assumptions of time-varying dependencies

In terms of the assumptions present in the transfer network, while incorporating expert knowledge can help improve the explanatory power of the model, some of the dependencies may not be robust. Due to the performance in the above sensitivity analysis and the controversial views from existing literature, the paper sets up a reverse time arc between physical health and mental health, along with removing the time arc between physical health and social health in the transfer network (shown in Fig. 4 in Appendix 3) to gain a new dynamic Bayesian network. The following parameter learning results are shown in Appendix 3 (Tables 12, 13, 14 and 15). The results show that the inverse time-varying dependencies between physical health and mental health is significantly weaker. Besides, other time-varying dependencies are similar.

## Conclusion and discussion

Research on healthy aging has become increasingly important due to the accelerating aging process and the emergence of issues related to older adults. This paper contributes to the literature by constructing an integrated research framework simultaneously incorporating multidimensional health, life satisfaction, and health behaviors of older adults aged 60 years and over to identify the time-varying dependencies between the factors. To the best of our knowledge, this paper is the first to study aging using BN and DBN, which overcomes several limitations of regression model. By structure learning of BN, the dependencies among factors related to healthy aging are found, contributing to a more comprehensive understanding of the pathways to achieving healthy aging. Moreover, dynamic Bayesian network is developed to capture the dynamic dependencies among the factors related to healthy aging by investigating the concurrent effects, one-period lagged effects and two-period lagged effects. Besides, the results of autoregressive effects present the predictive value of multidimensional health and life satisfaction of older adults aged 60 years and over. The findings jointly shed further insights on the time-varying features of healthy aging.

Many interesting conclusions are observed. First, in terms of different dimensions of health, mental health has a positive long-term effect on physical health. Self-rated health exhibits a positive long-term effect on mental health, while its impact on physical health is comparatively smaller in magnitude. Social health can promote current physical health and has lagged effects on physical and mental health, with the difference that the effect on physical health will accumulate over time while the effect on mental health will dissipate over time. Second, the contribution of health behaviors to health outcomes has been verified among older adults aged 60 years and over. Healthier behaviors have positive concurrent effects on mental health and social health, and it has long-term lagged effects on physical and mental health, which will accumulate over time. Third, health is verified by this paper as a determinant of life satisfaction among older adults aged 60 years and over. Self-rated health has positive concurrent and lagged effects on life satisfaction, and the lagged effects are also long-term and accumulate over time. Moreover, the autoregressive effects of life satisfaction and multidimensional health all proved to be positive, which illustrates the predictive value of current health and life satisfaction for future outcomes. Considering the deterioration of several health indicators in descriptive statistics, it further indicates that healthy aging is a comprehensive result arising from interactions of multiple factors. Finally, this paper finds that older adults aged 60 years and over with different socio-economic characteristics may have significant heterogeneity in life satisfaction, health, and health behaviors. The oldest old are in poorer physical condition than the younger old, but increased awareness of health may be a potential initiative to mitigate aging.

The above findings provide policy references for achieving healthy aging. Improving health status and life satisfaction is an important initiative to promote healthy ageing, requiring the dissemination of knowledge about healthy health behaviors among older adults aged 60 years and over as well as the encouragement of social participation and physical awareness. In addition, it found that long-term effects and accumulative effects are prevalent, thus policy guidance should be long-term, and policymakers should continuously guide older adults aged 60 years and over to develop healthier behaviors and actively improve their health and life satisfaction. Finally, policymakers should strengthen financial support for older adults aged 60 years and over and take gender differences, urban-rural differences, and the protective role of marriage into account when making decisions.

Despite the many strengths of this paper, there are still several limitations. First, the dataset has much missing data due to the death of respondents or loss of follow-up during the three periods, resulting in the final study sample being a bit small compared with the original sample (but similar situations have been observed in previous studies, and the sensitivity analysis shows the robustness of the findings). Second, there is a potential limitation of reducing external validity by dividing the responses on the likert scale into two groups for the sake of easier modeling and interpretation, which failed to gain more comprehensive conclusions. Third, incorporating additional factors potentially influencing healthy aging to the models can further complement the research framework. For example, more behavioral determinants of healthy aging can be incorporated to gain further insight into the benefits of lifestyles. Besides, considering the limitations of Bayesian networks in capturing only unidirectional dependencies, there may be potential bidirectional reciprocal relationships that cannot be found. Furthermore, the application of dynamic structure learning algorithms would be one of the extensions of this paper.

### Electronic supplementary material

Below is the link to the electronic supplementary material.

**Supplementary Material 1: Appendix 1.** Statement on the data preprocessing procedure. **Appendix 2.** Descriptive statistics grouped by gender. **Appendix 3.** Statement on the assumptions of transfer network. **Appendix 4.** Questions about mental health

## Data Availability

The CLHLS data is available at Peking University Open Research Data Platform, i.e., https://opendata.pku.edu.cn/dataverse/CHADS.

## Footnotes

1 Data is publicly available at the Peking University Open Research Data (http://opendata.pku.edu.cn/dataverse/CHADS).

2 Thanks for the support of GeNIe software: https://support.bayesfusion.com/docs/GeNIe/.
