# Supplementary material for: Enhancing understanding of healthy aging based on time-varying dependencies among multidimensional health, life satisfaction, and health behaviors of older adults aged 60 years and over
Source: BMC Public Health. 2024 Jan 16;24:192. doi: 10.1186/s12889-024-17752-2 (PMC10790531; doi:10.1186/s12889-024-17752-2)
Supplement: Supplementary file 1 — Supplementary Material 1: Appendix 1. Statement on the data preprocessing procedure. Appendix 2. Descriptive statistics grouped by gender. Appendix 3. Statement on the assumptions of transfer network. Appendix 4. Questions about mental health [file 12889_2024_17752_MOESM1_ESM.docx]

# Appendix 1. Statement on the data preprocessing procedure

As stated in the manuscript, a total of 9,765 respondents who were interviewed in 2011-2012 are employed as our initial sample, in which 3699 and 3163 samples are respectively dropped due to death or loss to follow-up in the 2014 and 2018 waves. In addition, considering missing data on the concerned variables, we removed 1,141 samples, resulting in a total of 1,762 samples as the final analytic sample. The paper aims to investigate time-varying dependencies between variables (concurrent effects, one-period lagged effects and two-period lagged effects), in which survival samples over three periods are required. Therefore, sample exclusion due to death or loss of access during the study period is necessary. Of the remaining 2903 samples, 837 samples lacked key information about mental health, subjective self-rated health, and self-perceived quality of life that had to be answered by participants in person. The key information is highly heterogeneous to individuals, which was additionally marked in the questionnaires to ensure that participants had to answer them in person. These samples with missing key information are suggested to be dropped because any data imputation method cannot obtain accurate estimates as well.

(1) Considering the potential problem of inconsistent estimates arising from the exclusion of the other 304 samples, we employ a significance test about the difference between analytic sample (including 1762 samples) and the sample without missing key information (including 2066 samples). The significance test is conducted between the two samples for the initial indicators of all variables, where t-test is employed for age and d12 which are continuous, and chi-square test is employed for the other indicators which are discrete. Based on R, the results of significance test are shown in Table 1.

**Table 1 Results of significance test**

| **Variables** | **Chi-squared/t value** | **p-value** |
| --- | --- | --- |
| **gender** | 0.3266 | 0.5677 |
| **Residence** | 0.6777 | 0.7126 |
| **age** | 0.2078 | 0.8354 |
| **SATIS** | 0.2654 | 0.9919 |
| **SELF** | 0.0367 | 0.9998 |
| **MENTAL** |  |  |
| look on the bright side of things | 0.0547 | 0.9996 |
| keep my belongings neat and clean | 0.5661 | 0.9668 |
| feel fearful or anxious | 0.2266 | 0.9940 |
| feel lonely and isolated | 0.2366 | 0.9935 |
| make own decision | 0.2196 | 0.9944 |
| feel useless with age | 0.5214 | 0.9714 |
| be happy as younger | 0.0798 | 0.9992 |
| **HEALTH BEHAVIORS** |  |  |
| how often eat fresh fruit? | 0.0377 | 0.9981 |
| how often eat vegetables? | 0.0990 | 0.9920 |
| exercise or not at present? | 0.4847 | 0.4863 |
| **SOCIAL** |  |  |
| do you play cards/mah-jongg at present? | 0.1951 | 0.9955 |
| do you take part in some social activities at present? | 0.2457 | 0.9930 |
| # of times traveling beyond home county/Residence in the past two years | 0.1295 | 0.8970 |
| **IADL** |  |  |
| able to go outside to visit neighbors? | 0.3037 | 0.8591 |
| able to go shopping by yourself? | 0.2853 | 0.8671 |
| able to make food by yourself? | 0.0245 | 0.9878 |
| able to wash clothes by yourself? | 0.0618 | 0.9696 |
| able to walk one kilometer? | 0.1947 | 0.9072 |
| able to carry 5kg weight? | 0.1975 | 0.9060 |
| able to crouch and stand three times? | 0.0500 | 0.9753 |
| able to take public transportation? | 0.1577 | 0.9242 |

As is shown, there is no statistically significant difference between the two samples.

(2) To gain a more robust conclusion, multiple imputation of missing values is conducted based on random forests despite the potential errors in the key information obtained from the data imputation methods. Based on the imputed 2903 samples, the structure learning results of Bayesian network are shown in **Fig. 1**. Compared to the original conclusion, there are only two paths have changed, which are the dependencies between physical health and mental health, and the dependencies between physical health and social health. Considering the fact that there are already two perspectives in the academia explaining the relationship between physical and mental health, i.e., top-down model and bottom-up model, there may be a bidirectional reciprocal relationship between physical health and mental health, as well as between physical health and social health. Moreover, the overall pathway from health behavior to multidimensional health to life satisfaction is not changed.

**Fig. 1 Structure learning results**

*Note:* The Bayesian network structure is learned based on PC algorithm, and the level of significance is set as the value 0.01 for the more robust learning process

In addition, transfer network (shown in **Fig. 2**) is also built to develop the dynamic Bayesian network based on the above network. Due to the changed paths in above network, the time arc between physical health and mental health and the time arc between physical health and mental health are removed, followed by gaining the results of parameter learning (shown in Table 2-4).

**Fig. 2 Transfer network**

*Note:* Considering the two pathways are not robust, the time arc between physical health and mental as well as time arc between physical health and social health are also removed.

**Table 2 Time-varying effects of self-rated health on physical health, mental health, and life satisfaction**

| Self-rated health |  | Physical health (t) | Mental health (t) | Satisfaction (t) |
| --- | --- | --- | --- | --- |
|  | no change | 38.25% | 28.26% | 96.40% |
| Self-rated health | t-1 | 38.99% (2.62%) | 28.56% (1.06%) | 97.05% (0.67%) |
|  | t-2 | 38.31% (0.16%) | 28.50% (0.85%) | 97.07% (0.70%) |

**Table 3 Time-varying effects of health behaviors on physical, mental health, and social health**

| Health behaviors |  | Physical health (t) | Mental health (t) | Social health (t) |
| --- | --- | --- | --- | --- |
|  | no change | 38.25% | 28.26% | 22.20% |
| Health behaviors | t-1 | 43.11% (12.71%) | 39.92% (41.26%) |  |
|  | t-2 | 41.71% (9.05%) | 36.16% (27.95%) |  |

**Table 4 Time-varying effects of social health on physical health and mental health**

| Social health |  | Mental health (t) |
| --- | --- | --- |
|  | no change | 28.26% |
| Social health | t |  |
|  | t-1 | 33.03% (16.88%) |
|  | t-2 | 33.71% (19.29%) |

As is shown in the above tables, the directions of time-varying dependencies are unchanged. In addition, two-period lagged effects are generally significant, and several two-period lagged effects are stronger than one-period lagged effects or close to them, suggesting the long-term and accumulative effects are still found. For the robustness, the origin transfer network (**Fig. 3**) is also employed to develop the dynamic Bayesian network. The results of parameter learning are shown in Table5-7.

**Fig. 3 Origin transfer network**

*Note:* The origin transfer network is also employed to develop the dynamic Bayesian network.

**Table 5 Time-varying effects of self-rated health on physical health, mental health, and life satisfaction**

| Self-rated health |  | Physical health (t) | Mental health (t) | Satisfaction (t) |
| --- | --- | --- | --- | --- |
|  | no change | 42.57% | 31.24% | 96.40% |
| Self-rated health | t-1 | 42.74% (0.40%) | 30.71% (-1.70%) | 97.05% (0.67%) |
|  | t-2 | 42.65% (0.19%) | 30.78% (-1.47%) | 97.07% (0.70%) |

**Table 6 Time-varying effects of health behaviors on physical, mental health, and social health**

| Health behaviors |  | Physical health (t) | Mental health (t) |
| --- | --- | --- | --- |
|  | no change | 42.57% | 31.24% |
| Health behaviors | t-1 | 46.82% (9.98%) | 41.69% (33.45%) |
|  | t-2 | 45.23% (6.25%) | 39.19% (25.45%) |

**Table 7 Time-varying effects of social health on physical health and mental health**

| Social health |  | Mental health (t) |
| --- | --- | --- |
|  | no change | 31.24% |
| Social health | t-1 | 36.72% (17.54%) |
|  | t-2 | 37.32% (19.46%) |

All conclusions are generally similar.

# Appendix 2. Descriptive statistics grouped by gender

**Table 8** **Descriptive statistics grouped by gender during the study period.**

| All | | | | | |
| --- | --- | --- | --- | --- | --- |
| Variable | Category | man | | woman | |
|  |  | Frequency | Proportion | Frequency | Proportion |
| Residence | 0 | 2336 | 86.90% | 2154 | 82.91% |
|  | 1 | 352 | 13.10% | 444 | 17.09% |
| Age | 1 | 1625 | 60.45% | 1328 | 51.12% |
|  | 2 | 796 | 29.61% | 907 | 34.91% |
|  | 3 | 229 | 8.52% | 286 | 11.01% |
|  | 4 | 38 | 1.41% | 77 | 2.96% |
| SATIS | 0 | 91 | 3.39% | 89 | 3.43% |
|  | 1 | 2597 | 96.61% | 2509 | 96.57% |
| Satisfaction | 0 | 274 | 10.19% | 391 | 15.05% |
|  | 1 | 2414 | 89.81% | 2207 | 84.95% |
| Self-rated health | 0 | 1831 | 68.12% | 1967 | 75.71% |
|  | 1 | 857 | 31.88% | 631 | 24.29% |
| Mental health | 0 | 2163 | 80.47% | 2123 | 81.72% |
|  | 1 | 525 | 19.53% | 475 | 18.28% |
| Health behaviors | 0 | 1787 | 66.48% | 1957 | 75.33% |
|  | 1 | 901 | 33.52% | 641 | 24.67% |
| Social health | 0 | 878 | 32.66% | 1455 | 56.00% |
|  | 1 | 1810 | 67.34% | 1143 | 44.00% |
| Physical health  Economic status | 1 | 270 | 10.04% | 313 | 12.05% |
|  | 2 | 1848 | 68.75% | 1799 | 69.25% |
|  | 3 | 570 | 21.21% | 486 | 18.71% |
| marriage | 0 | 777 | 28.91% | 1599 | 61.55% |
|  | 1 | 1911 | 71.09% | 999 | 38.45% |

**Table 9 Descriptive statistics grouped by gender in 2011**

| Year=2011 | | | | | |
| --- | --- | --- | --- | --- | --- |
| Variable | Category | man | | woman | |
|  |  | Frequency | Proportion | Frequency | Proportion |
| Residence | 0 | 782 | 87.28% | 723 | 83.49% |
|  | 1 | 114 | 12.72% | 143 | 16.51% |
| Age | 1 | 674 | 75.22% | 564 | 65.13% |
|  | 2 | 175 | 19.53% | 239 | 27.60% |
|  | 3 | 41 | 4.58% | 42 | 4.85% |
|  | 4 | 6 | 0.67% | 21 | 2.42% |
| SATIS | 0 | 45 | 5.02% | 43 | 4.97% |
|  | 1 | 851 | 94.98% | 823 | 95.03% |
| Satisfaction | 0 | 83 | 9.26% | 131 | 15.13% |
|  | 1 | 813 | 90.74% | 735 | 84.87% |
| Self-rated health | 0 | 576 | 64.29% | 634 | 73.21% |
|  | 1 | 320 | 35.71% | 232 | 26.79% |
| Mental health | 0 | 717 | 80.02% | 686 | 79.21% |
|  | 1 | 179 | 19.98% | 180 | 20.79% |
| Health behaviors | 0 | 573 | 63.95% | 618 | 71.36% |
|  | 1 | 323 | 36.05% | 248 | 28.64% |
| Social health | 0 | 194 | 21.65% | 399 | 46.07% |
|  | 1 | 702 | 78.35% | 467 | 53.93% |
| Physical health  Economic status | 1 | 104 | 11.61% | 142 | 16.40% |
|  | 2 | 625 | 69.75% | 560 | 64.67% |
|  | 3 | 167 | 18.64% | 164 | 18.94% |
| marriage | 0 | 223 | 24.89% | 474 | 54.73% |
|  | 1 | 673 | 75.11% | 392 | 45.27% |

**Table 10 Descriptive statistics grouped by gender in 2014**

| Year=2014 | | | | | |
| --- | --- | --- | --- | --- | --- |
| Variable | Category | man | | woman | |
|  |  | Frequency | Proportion | Frequency | Proportion |
| Residence | 0 | 782 | 87.28% | 724 | 83.60% |
|  | 1 | 114 | 12.72% | 142 | 16.40% |
| Age | 1 | 579 | 64.62% | 472 | 54.50% |
|  | 2 | 244 | 27.23% | 293 | 33.83% |
|  | 3 | 63 | 7.03% | 76 | 8.78% |
|  | 4 | 10 | 1.12% | 25 | 2.89% |
| SATIS | 0 | 25 | 2.79% | 19 | 2.19% |
|  | 1 | 871 | 97.21% | 847 | 97.81% |
| Satisfaction | 0 | 88 | 9.82% | 138 | 15.94% |
|  | 1 | 808 | 90.18% | 728 | 84.06% |
| Self-rated health | 0 | 597 | 66.63% | 658 | 75.98% |
|  | 1 | 299 | 33.37% | 208 | 24.02% |
| Mental health | 0 | 721 | 80.47% | 714 | 82.45% |
|  | 1 | 175 | 19.53% | 152 | 17.55% |
| Health behaviors | 0 | 574 | 64.06% | 649 | 74.94% |
|  | 1 | 322 | 35.94% | 217 | 25.06% |
| Social health | 0 | 241 | 26.90% | 453 | 52.31% |
|  | 1 | 655 | 73.10% | 413 | 47.69% |
| Physical health  Economic status | 1 | 69 | 7.70% | 95 | 10.97% |
|  | 2 | 649 | 72.43% | 628 | 72.52% |
|  | 3 | 178 | 19.87% | 143 | 16.51% |
| marriage | 0 | 239 | 26.67% | 522 | 60.28% |
|  | 1 | 657 | 73.33% | 344 | 39.72% |

**Table 11 Descriptive statistics grouped by gender in 2018**

| Year=2018 | | | | | |
| --- | --- | --- | --- | --- | --- |
| Variable | Category | man | | woman | |
|  |  | Frequency | Proportion | Frequency | Proportion |
| Residence | 0 | 772 | 86.16% | 707 | 81.64% |
|  | 1 | 124 | 13.84% | 159 | 18.36% |
| Age | 1 | 372 | 41.52% | 292 | 33.72% |
|  | 2 | 377 | 42.08% | 375 | 43.30% |
|  | 3 | 125 | 13.95% | 168 | 19.40% |
|  | 4 | 22 | 2.46% | 31 | 3.58% |
| SATIS | 0 | 21 | 2.34% | 27 | 3.12% |
|  | 1 | 875 | 97.66% | 839 | 96.88% |
| Satisfaction | 0 | 103 | 11.50% | 122 | 14.09% |
|  | 1 | 793 | 88.50% | 744 | 85.91% |
| Self-rated health | 0 | 658 | 73.44% | 675 | 77.94% |
|  | 1 | 238 | 26.56% | 191 | 22.06% |
| Mental health | 0 | 725 | 80.92% | 723 | 83.49% |
|  | 1 | 171 | 19.08% | 143 | 16.51% |
| Health behaviors | 0 | 640 | 71.43% | 690 | 79.68% |
|  | 1 | 256 | 28.57% | 176 | 20.32% |
| Social health | 0 | 443 | 49.44% | 603 | 69.63% |
|  | 1 | 453 | 50.56% | 263 | 30.37% |
| Physical health  Economic status | 1 | 97 | 10.83% | 76 | 8.78% |
|  | 2 | 574 | 64.06% | 611 | 70.55% |
|  | 3 | 225 | 25.11% | 179 | 20.67% |
| marriage | 0 | 315 | 35.16% | 603 | 69.63% |
|  | 1 | 581 | 64.84% | 263 | 30.37% |

# Appendix 3. Statement on the assumptions of transfer network

In terms of the assumptions present in the transfer network, while incorporating expert knowledge can help improve the explanatory power of the model, some of the dependencies may not be robust. Due to the performance in the above sensitivity analysis (Appendix1) and the controversial views from existing literature, the paper sets up a reverse time arc between physical health and mental health, along with removing the time arc between physical health and social health in the transfer network (**Fig. 4**) to gain a new dynamic Bayesian network. The following parameter learning results are shown in Table 12-15. The results show that the inverse time-varying dependencies between physical health and mental health is significantly weaker. Besides, other time-varying dependencies are similar.

**Fig. 4 Transfer network for sensitivity analysis**

*Note:* The time arc between physical health and mental health is reversed, and the time arc between physical health and social health is removed.

**Table 12 Time-varying effects of mental health on physical health**

| Multidimensional health |  | mental health(t) |
| --- | --- | --- |
|  | no change | 36.81% |
| Physical health | t-1 | 37.24% (1.09%) |
|  | t-2 | 37.73% (2.50%) |

**Table 13 Time-varying effects of self-rated health on physical health, mental health, and life satisfaction**

| Self-rated health |  | Physical health (t) | Mental health (t) | Satisfaction (t) |
| --- | --- | --- | --- | --- |
|  | no change | 47.91% | 36.81% | 96.05% |
| Self-rated health | t-1 | 48.06% (0.31%) | 36.08% (-1.98%) | 96.69% (0.67%) |
|  | t-2 | 48.01% (0.21%) | 36.29% (-1.41%) | 96.81% (0.79%) |

**Table 14 Time-varying effects of health behaviors on physical, mental health, and social health**

| Health behaviors |  | Physical health (t) | Mental health (t) |
| --- | --- | --- | --- |
|  | no change | 47.91% | 36.81% |
| Health behaviors | t-1 | 50.15% (4.68%) | 45.22% (22.85%) |
|  | t-2 | 49.30% (2.90%) | 45.38% (23.28%) |

**Table 15 Time-varying effects of social health on physical health and mental health**

| Social health |  | Mental health (t) |
| --- | --- | --- |
|  | no change | 28.26% |
| Social health | t-1 | 42.20% (49.33%) |
|  | t-2 | 42.00% (48.62%) |

# Appendix 4. Questions about mental health

**Table 16 Questions about mental health**

| Questions | Answers |
| --- | --- |
| Do you always look on the bright  side of things? | 1=always, 2=often, 3=sometimes, 4=seldom, 5=never |
| Do you like to keep your belongings  neat and clean? | 1=always, 2=often, 3=sometimes, 4=seldom, 5=never |
| Do you feel energized？ | 1=always, 2=often, 3=sometimes, 4=seldom, 5=never |
| Have you been ashamed, regretful, or  felt guilty about things you've done? | 1=always, 2=often, 3=sometimes, 4=seldom, 5=never |
| Are you angry at people or things you  don't like around you? | 1=always, 2=often, 3=sometimes, 4=seldom, 5=never |
| Can you make your own decisions  concerning your personal affairs? | 1=always, 2=often, 3=sometimes, 4=seldom, 5=never |
| Do you feel that people around you are  not trustworthy? | 1=always, 2=often, 3=sometimes, 4=seldom, 5=never |
